# Exploring the arthritogenicity of *Streptococcus dysgalactiae* subspecies *equisimilis*

**DOI:** 10.1186/s12866-018-1160-5

**Published:** 2018-02-27

**Authors:** Oddvar Oppegaard, Haima Mylvaganam, Steinar Skrede, Bård Reiakvam Kittang

**Affiliations:** 1 Department of Medicine, Haukeland University Hospital, 5021 Bergen, Norway; 2 Department of Clinical Science, University of Bergen, Bergen, Norway; 3 Department of Microbiology, Haukeland University Hospital, Bergen, Norway; 4 Department of Medicine, Haraldsplass Deaconess Hospital, Bergen, Norway

**Keywords:** *Streptococcus dysgalactiae* Subspecies *equisimilis*, Group C streptococcus, Group G streptococcus, Osteoarticular infections, Septic arthritis, Tissue tropism, Pilus, FCT-region, Collagen, Fibronectin

## Abstract

**Background:**

During the past decades, *Streptococcus dysgalactiae* subspecies *equisimilis* (SDSE) has been increasingly recognized as an important human pathogen. Osteoarticular infections is one of the predominant disease manifestations of SDSE, but the pathogenetic rationale for its arthritogenicity has yet to be unravelled. We aimed to explore if the rising incidence of osteoarticular infections caused by this pathogen in our region emanated from clonal expansion of strains with enhanced tropism for bone and joint tissue components or orthopaedic implants.

**Results:**

Twenty-nine SDSE-isolates associated with osteoarticular infections were retrospectively identified. Their genomic content and affinity for fibronectin, collagen and stainless steel were compared to 24 temporally and geographically matched SDSE blood culture isolates obtained from patients without bone or joint infections.

Despite a thorough genetic and phenotypic dissection, neither the presence or absence of any single gene, nor the binding abilities of the SDSE isolates, were predictive of clinical entity. SNP analysis revealed a heterogenous population, and a correlation between phylogenetic relationships and disease manifestation was not evident. However, we identified a strong concordance between phenotypic binding abilities and genetic variations in the pilus-region, also denoted as the FCT-region (**F**ibronectin binding, **C**ollagen binding and **T**-antigen). This observation could be related to the ample and varied repertoire of putative adhesins residing within this region, including proteins predicted to adhere to fibronectin and collagen, as well as fibrinogen.

**Conclusions:**

SDSE strains associated with osteoarticular infections do not emanate from subpopulation characterized by distinct genetic or phenotypic traits. The genetic architecture of the pilus region was predictive of the adhesive properties of the SDSE-isolates, but its role in tissue tropism needs further investigation. To the best of our knowledge, this is the first comprehensive characterization of the genetic landscape of the SDSE pilus region.

**Electronic supplementary material:**

The online version of this article (10.1186/s12866-018-1160-5) contains supplementary material, which is available to authorized users.

## Background

Osteoarticular infections (OAIs) cause considerable morbidity and mortality worldwide, and streptococci are second only to staphylococci as causative agents [1–3]. *Streptococcus dysgalactiae* subspecies *equisimilis* (SDSE), a β-haemolytic streptococcus predominantly possessing the Lancefield group C or G antigen, appears to have a tropism for bone and joints, and the incidence of OAIs caused by SDSE has increased significantly over the past decades [4]. This pathogen preferentially targets the elderly and people with comorbidities, and local or systemic risk factors are present in more than 90% of the patients with OAIs caused by SDSE [4, 5]. Inasmuch as the prevalence of both chronic maladies and orthopaedic implants is rising, the upsurge of SDSE OAIs is likely to continue [6, 7]. Of further concern, such SDSE infections appear to be associated with an unfavourable clinical outcome compared to OAIs caused by other streptococci [8]. The pathogenetic rationale for the arthritogenicity of SDSE, however, has yet to be elucidated.

Bacterial adhesion to bone and joint tissues is a pivotal step in the pathogenesis of OAIs [9]. Fibronectin and collagen (type I and II) are among the main constituents of these tissues, and have been demonstrated to be important targets for bacterial attachment [10, 11]. Moreover, fibronectin-binding proteins appear to be implicated in the adherence of *Staphylococcus aureus* to orthopaedic implants [12]. Knowledge on adhesive determinants in SDSE is scarce, but a few fibronectin binding proteins have been identified, including the M-protein, encoded by the *emm*-gene [13, 14]. In the genetically closely related *Streptococcus pyogenes* (*S. pyogenes*), the adhesin Cpa has been shown to mediate attachment to tissues containing collagen type I. Furthermore, patients with a recent history of septic arthritis or osteomyelitis caused by *S. pyogenes* had increased anti-Cpa-titers, suggesting a role for this virulence factor in the pathogenesis of OAIs [15]. Cpa is part of the pilus-apparatus in *S. pyogenes*, encoded by the FCT-region (**F**ibronectin binding, **C**ollagen binding and **T**-antigen). This region displays a substantial genetic heterogeneity, and nine different FCT-variants have been characterized [16]. In a recent effort to delineate *S. pyogenes* strains according to their tropism for skin or throat, the FCT-region and the *emm*-region were identified as two of the most likely determinants [17]. Homologues of both these regions are present in SDSE, and a correlation to their propensity for OAIs is conceivable.

We wished to investigate if the increasing incidence of SDSE OAIs in our region emanated from a bacterial subpopulation with enhanced tropism for bone and joint components or orthopaedic implants. The affinity of clinical SDSE isolates to fibronectin, collagen and metal surfaces were explored. Furthermore, we employed whole genome sequencing (WGS) to correlate the genetic repertoire of adhesins with clinical manifestations and phenotypic binding properties, with particular emphasis on the FCT- and *emm*-regions. To reduce the number of confounding factors, we included a control group of temporally and geographically matched SDSE blood culture isolates obtained from patients with local risk factors for OAI, but without bone or joint infections.

## Results

From a total of 42 clinical OAI cases, 29 bacterial isolates were available for analysis; 16 were obtained from blood cultures and 13 detected in synovial fluid or tissue specimens. The control group included 24 cases associated with SDSE bacteraemia, comprising 19 skin and soft-tissue infections, four patients with pneumonia and one case of necrotizing soft-tissue infection. The main clinical characteristics of the two groups are summarized in Table 1. The patients in the control group were slightly older, and had a higher burden of systemic comorbidities than patients with OAIs. The presence of orthopaedic implants was the most common local risk factor in both groups, whereas recent surgical procedures or previous septic arthritis of the affected joint were overrepresented in the OAI group.

**Table 1:** Clinical characteristics of the patient cohorts

Clinical characteristic	OAI *n* = 29		Non-OAI *n* = 24		*P-value*
Demography					
Age, median (IQR)	67	(49–81)	80	(65–89)	0.06
Male sex	20	(68%)	8	(33%)	**0.01**
Systemic comorbidity					
CCI, median (IQR)	1	(0–3)	2	(1–4)	**0.004**
Chronic organ failure	10	(34%)	12	(50%)	0.28
Diabetes	4	(14%)	3	(13%)	1.0
Active Malignancy	3	(10%)	5	(21%)	0.44
Immunosuppression	3	(10%)	7	(29%)	0.16
Local risk factors					
Orthopaedic implant	9	(31%)	16	(67%)	**0.01**
Pre-existing joint disease	5	(17%)	10	(42%)	0.07
Surgical proc. < 4 weeks	7	(24%)	1	(4%)	0.06
Previous septic arthritis	8	(28%)	2	(8%)	0.09

*OAI* Osteoarticular infection, *CCI* Charlson comorbidity Index, *IQR* Inner quartile range; Surgical proc. < 4 weeks, invasive joint procedure performed within 4 weeks prior to admission. Chronic organ failures included any of cardiac, renal, respiratory and hepatic failures. All data presented as *n* (% of cases), except Age and CCI, presented as median (IQR). Significant *P*-values are highlighted in bold

### Whole genome sequencing, genotyping and phylogenetic relationships

The assembled genomes had an average genome size of 2.1 Mbp, the mean coverage was ~ 80 fold, and the G + C-content was approximately 39%. Genotyping revealed a diverse population, comprising 12 *emm*-types in total, and 11 and nine different *emm*-types were represented among the OAIs and non-OAIs, respectively. *stG485*, *stG643* and *stG6* were most frequently encountered in both groups, and significant associations between *emm*-type and clinical manifestations were not detected. Twenty-six multilocus sequence typing (MLST)-profiles were identified. A strong concordance between *emm*-type and MLST-profile was noted except for *stg485*; an apparently promiscuous *emm*-type associated with nine different MLST-profiles. Comparisons of the entire *emm*-genes of these *stG485* isolates revealed great heterogenicity in the 5′-terminal part, indicative of gene recombination events. The *emm*-type, MLST-profile, binding abilities and clinical manifestation associated with each isolate is summarized in Additional file 1: Table S1.

**Fig. 1:**
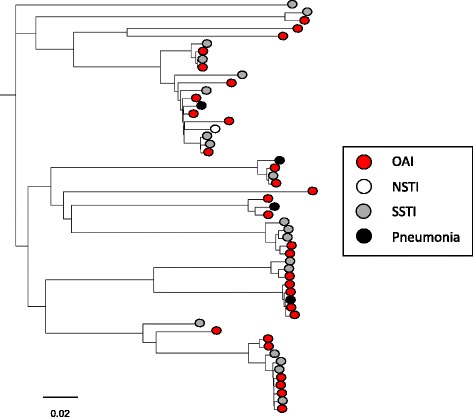
Phylogenetic relationship of SDSE isolates associated with different clinical manifestations. Phylogenetic tree based on SNP-analysis of the 53 SDSE genomes. Clinical manifestation does not appear to be associated with particular phylogenetic clades. OAI, osteoarticular infection; NSTI, necrotizing soft-tissue infection; SSTI, skin and soft-tissue infection. Scale indicates substitutions per site. The phylogenetic tree was constructed in Geneious by global alignment with free end gaps using the Tamura-Nei genetic distance model [38]. FastTree was used for visualising and tree-rendering [39]

From a core genome of 1.72 Mbp, a total of 25,365 single nucleotide polymorphisms (SNPs) valid in all genomes were identified. A substantial genetic heterogenicity was evident, and a relationship between particular phylogenetic clades and clinical manifestations could not be identified. On the contrary, strains associated with OAI and non-OAI were often genetically closely related, and in some instances differed by less than 30 SNPs. A phylogenetic tree based on the SNP-analysis is depicted in Fig. 1.

### Screening for adhesive determinants

The repertoire of adhesive determinants detected in the SDSE-isolates is presented in Table 2. The fibronectin binding genes *gapC*, *lmb*, *fbp54* and *shr* were present in all 53 isolates, and displayed an inter-strain homology of 99%. Conversely, *gfba* was detected in only 28 isolates (53%). Its presence correlated strongly with *emm*-type, but was not significantly associated with disease category, *P* = 0.6. Eight of the *gfba* genes translated into truncated proteins, whereof five originated from SDSE-isolates associated with non-OAIs. The *fnB*-gene was ubiquitous in the SDSE isolates, and two differently sized variants were identified. The signal peptides and the C-terminal parts of their predicted proteins were conserved, but the larger of the two variants contained an additional fragment of 359 amino acid residues in the N-terminal region, bearing resemblance to the stalk-like B-region of the collagen binding protein Cna in *Staphylococcus aureus* (protein family 05738). The *fnB*-variants have been deposited in GenBank under the accession numbers KX838908 (*fnB* small variant), KX838909 (*fnB* large variant). Neither of the two variants were significantly associated with OAI.

**Table 2:** Repertoire of adhesive determinants in the SDSE-isolates

Gene	Reference Accession number	OAI *n* = 29		Non-OAI *n* = 24		Rep. SDSE
*emm - gene*	BAH80747	29	(100)	24	(100)	+
FCT-regions						
FCT_1_	BAH80674–79	29	(100)	24	(100)	+
FCT_2_	BAH82260–65	26	(90)	22	(92)	+
Adhesins						
*prtF1/gfba*	U31115	14	(48)	14	(58)	+
*prtF2/fbaB/pfbp*	AB084272/AY612221	0	(0)	0	(0)	–
*fnB*	BAH82466	29	(100)	24	(100)	+
*fbaA*	AB040536	0	(0)	0	(0)	+
*sof*	X83303	0	(0)	0	(0)	–
*sfbX*	AF335322	0	(0)	0	(0)	–
*gapdh/gapC*	AF375662	29	(100)	24	(100)	+
*fbp54*	BAH81758	29	(100)	24	(100)	+
*shr*	BAH82346	29	(100)	24	(100)	+
*lmb*	BAH81434	29	(100)	24	(100)	+
*sclA*	AY459361	0	(0)	0	(0)	–

All data presented as *n* (% of cases). *OAI* Osteoarticular infection, *Rep. SDSE* Previously reported in SDSE. The symbols + and – denote presence and absence of genetic feature, respectively

Lastly, we performed genome wide comparisons on SDSE isolates associated with the two clinical groups, attempting to identify genes potentially associated with OAIs. Notably, no single gene was found to be predictive of disease manifestation.

### Characterization of the FCT-regions

**Fig. 2:**
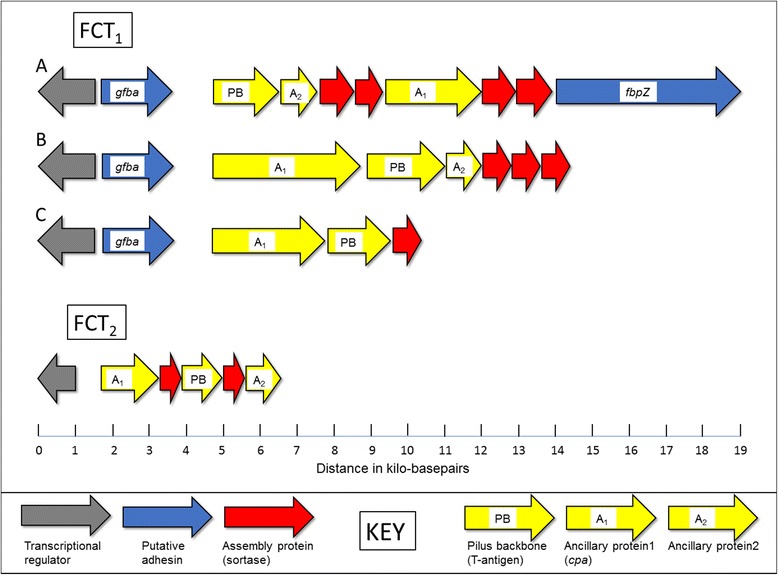
Characterization of the fibronectin and collagen binding T-antigen (FCT) regions. The vast majority of the SDSE-isolates harboured two FCT-regions, denoted FCT_1_ and FCT_2_, respectively. Three structurally different variants of the FCT_1_-region were detected, designated A, B and C. Of these, variant A was identified in 51 out of 53 isolates, although some variations were observed; the fibronectin binding protein Gfba was absent in 24 isolates, whereas Ancillary protein 1 along with the two downstream Sortase enzymes were absent in 11 isolates. The FCT_1_ variants A, B and C detected in our SDSE isolates resembled the *S. pyogenes* variants FCT-6, FCT-5 and FCT-1, respectively.^15^ The FCT_2_ region had a conserved genetic composition, but was absent in five isolates. The figure is drawn to scale

The genetic organization of the SDSE-isolates is presented in Fig. 2. The majority of the isolates contained two separate FCT-regions, herein denoted FCT_1_ and FCT_2_, but in five isolates the FCT_2_-region was absent. In all but two isolates, FCT_1_ had a structural composition resembling the FCT-6 variant described in *S. pyogenes* [16]. Some variations were observed, though, including loss of *cpa* in 11 strains and *gfba* in 24 strains. Two SDSE isolates, bearing the *stG120* and *stG652 emm*-types, had FCT_1_-regions homologous to the *S. pyogenes* variants FCT-1 and FCT-5, respectively. The genetic content of the FCT_2_-region was more conserved among the SDSE isolates, and resembled the FCT-2 variant reported in *S. pyogenes*. FCT_2_ encoded only the pilus construct, whereas the FCT_1_-region also encompassed putative adhesins in all the isolates, including the fibronectin-binding gene *gfba* and the putative collagen-binding pilus-tip adhesin, *cpa*. Furthermore, in 51 out of 53 isolates, FCT_1_ harboured a 5 kbp long gene homologous to *fbpZ* (GenBank accession number BAN92583) annotated as a fibronectin-binding gene in SDSE167 (GenBank accession number AP012976). Conserved Domain Blast of *fbpZ*, revealed the presence of an SdrG domain (protein family 10,425), demonstrated to facilitate fibrinogen binding in *Staphylococcus epidermidis* [18]. Moreover, it also contained fragments of domains involved in collagen binding (superfamily cl27951). However, apart from *gfba*, the binding properties of these SDSE homologues have not been investigated.

### Phenotypic binding assays

**Fig. 3:**
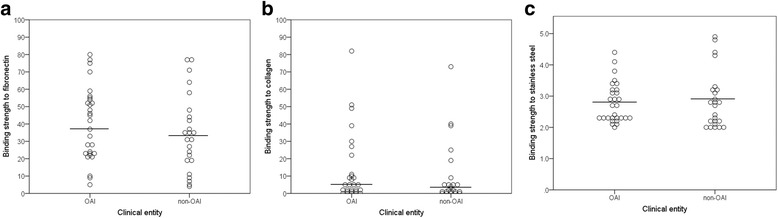
The phenotypic binding abilities of the SDSE isolates associated with OAIs and non-OAIs. The affinity of the SDSE isolates for fibronectin (panel **a**), collagen (panel **b**) and stainless steel (panel **c**) did not differ significantly between strains isolated from cases of OAIs and non-OAIs. All measurements were performed in triplicate and repeated thrice. The circles represent the mean value for each isolate. The horizontal lines indicate the median value for the clinical categories. The y-axis in Panel a and b indicate the adherence-level of the isolates as a percentage of the original inoculum. The y-axis in Panel c represents the measured binding strength of the isolates divided by the mean values obtained from control wells

The phenotypic binding properties of the SDSE isolates are presented in Fig. 3. In general, all the SDSE isolates bound significantly to fibronectin. The measured adherence was evenly distributed, ranging from 4% to 80% of the original inoculum, and specific subpopulations were not discernible. The median adherence level did not differ significantly between the OAI isolates (median 37%, IQR 23–54%) and the non-OAI isolates (median 33%, IQR 19–45%), *P* = 0.39. Similarly, phenotypic binding to fibronectin did not correlate with the presence of *gfba* or with the two different variants of *fnB*. However, a concordance with *emm*-type and MLST-profile was noted, except for isolates of genotype *stG485*.

The binding of all SDSE isolates to collagen II mirrored that to collagen I, only slightly weaker (Additional file 1: Table S1). Moreover, inhibition assays performed by preincubating SDSE-isolates with increasing concentrations of soluble collagen I, demonstrated a reduction in SDSE binding to collagen I as well as collagen II in a dose dependent manner (data not shown). Preincubation with collagen II had a similar effect. Taken together, this infers a common binding site for these two collagen types, and only data for collagen I are presented below. Ten isolates did not have any measurable interaction with collagen, and only 26 out of 53 showed > 5% binding. Two isolates belonging to the genotype *stG10*, and one isolate harbouring the *stG120 emm*-gene displayed a very strong adherence to collagen; 51%, 73% and 82% of the original inoculum, respectively. The affinity for collagen was relatively similar among OAI isolates (median 5%, IQR 1–11%) and non-OAI isolates (median 3%, IQR 1–6%), *P* = 0.38.

No isolates were classified as weak or non-binders in the stainless-steel assay. Six isolates were defined as strong binders; four of them belonging to genotype *stG6*, one to *stG485*, and one to *stG652*. The remaining 47 isolates were categorized as having medium binding ability. Adherence to stainless-steel did not differ significantly between OAI and non-OAI-associated isolates. Similarly, a focused analysis on the subgroup of isolates obtained from patients with orthopaedic implants, did not reveal any significant association between stainless steel adherence and orthopaedic implant infections. The gene *gfba* was identified in all the six isolates displaying strong binding ability to stainless steel, but this was not a general phenotypic trait of isolates harbouring *gfba*.

### Genetic variations in FCT-regions and phenotypic binding abilities

Since no single genes were predictive of the SDSE binding abilities, we speculated that the bacterial adhesive potential emanated from interplay between several genes in the FCT-regions. Small genetic variation leading to different domain functions might also be contributory, but such differences could be difficult to detect. Accordingly, we constructed phylogenetic trees of the FCT region, based on concatenation of the predicted protein sequences, excluding proteins primarily involved in the pilus assembly. The SDSE isolates were dichotomized into high or low binding strains to facilitate comparison to phylogenetic relationships. The isolates were divided into two approximately equally sized groups, using arbitrary cut off values of ≥5% and ≥30% for collagen and fibronectin, respectively.

**Fig. 4:**
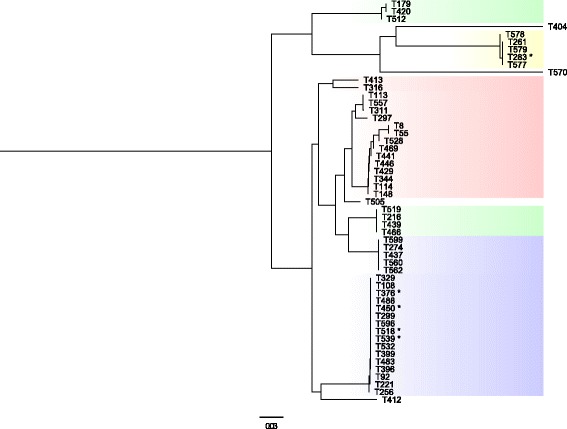
Phylogenetic tree of the FCT_1_-region and its relation to phenotypic binding abilities. The phenotypic binding abilities of the SDSE isolates concorded strongly with genetic variations in the FCT_1_-region. Based on their affinity for fibronectin, collagen and stainless steel, the isolates could be segregated into four distinct categories. Isolates highlighted in green adhered strongly to fibronectin and collagen, whereas isolates highlighted in yellow bound poorly to both substances. The blue marking signifies high-level binding to fibronectin and a low affinity for collagen. Inversely, the isolates highlighted in the red adhered well to collagen, but not to fibronectin. The isolates highlighted in yellow had a strong affinity for stainless steel, whereas the other isolates were characterized by a medium binding ability to stainless steel. Isolates marked with an asterisk had phenotypic binding characteristics deviating from their colour-coded category. Four isolates were phylogenetic outliers. Scale indicates substitutions per site. The phylogenetic tree was constructed in Geneious by global alignment with free end gaps using the Jukes-Cantor genetic distance model [38]. FastTree was used for visualising and tree-rendering [39]

Notably, the phylogenetic tree of the FCT_1_-region grouped the isolates in high concordance with their affinity for fibronectin, collagen and even stainless steel. Only three, two and nil misclassifications were noted for these substances, respectively. Conversely, the FCT_2_-region did not reveal a similar discriminatory ability, and a correlation with clinical manifestation was not evident for either of the two FCT-regions. The phylogenetic tree of the FCT_1_-region is shown in Fig. 4.

## Discussion

Osteoarticular infections (OAIs) constitute one of the predominant clinical manifestations of invasive SDSE disease [19]. The pathogenetic mechanisms underpinning their tropism for bone and joints have not previously been explored, but adherence to different tissue components has been postulated to be of importance in other Gram-positive pathogens [10, 11, 15]. In the present study, OAI and non-OAI-associated SDSE isolates could neither be distinguished according to their adhesive properties nor their genetic repertoire. However, we found the phenotypic binding abilities of SDSE isolates to fibronectin, collagen and stainless steel to concord strongly with genetic variations in the FCT_1_-region. This observation could be related to the ample and varied repertoire of putative adhesins encoded by this region, including proteins predicted to adhere to fibronectin and collagen, as well as fibrinogen.

The FCT-region encodes the pilus-structures, and like *Streptococcus agalactiae*, our sample of SDSE-isolates was found to predominantly harbour two separate pilus-encoding regions [20]. The FCT_2_-region encompassed only the structural components of the pilus, and was neither correlated with clinical manifestations nor binding properties. Some isolates had even lost the entire FCT_2_-region. This feature has also been reported in *S. agalactiae*, and a role for the second pilus in xeno-specificity was suggested [20]. Lending support to this hypothesis, the strictly human pathogen *S. pyogenes* only harbours the FCT_1_-region, whereas both SDSE and *S. agalactiae* are associated also with animal infections.

In general, we found SDSE to adhere well to fibronectin, whereas attachment to collagen displayed greater variability. SDSE has previously been shown to confer high levels of binding to both fibronectin and collagen, but the collagen binding appeared to be trypsin-resistant, and the involvement of non-protein surface components in some strains is possible [21, 22]. Three of our SDSE isolates demonstrated a very high level of adherence to collagen, whereof two belonged to the *emm*-type *stG10*, and one to *stG120*. These *emm*-types encoded a consensus motif in the N-terminal hypervariable region of the M-protein, (A/T/E) XYLXX(L/F) N, reported to mediate strong binding to collagen and postulated to be involved in the development of acute rheumatic fever [23]. However, several of our SDSE isolates lacking this motif in their predicted M proteins also bound strongly to collagen, indicating that SDSE can employ multiple strategies for attachment to host surfaces.

Despite a broadly targeted molecular screening, we could not relate SDSE clinical manifestations to the presence, absence or mutation of any single gene or gene-variant, indicating that if genetic differences are present, they are likely very subtle. Indeed, SNP-analysis did not reveal strains associated with OAIs to emanate from a distinct phylogenetic clade. Similar conclusions have been drawn in studies on tissue tropism in *Staphylococcus aureus* [24]. Our findings are further corroborated by the diverse clinical manifestations produced by virtually identical isolates in clonal outbreaks of *S. pyogenes* and SDSE disease [25, 26]. However, exploring bacterial pathogens only at the DNA-level, precludes the consideration of transcriptional and translational regulatory events, and further studies are warranted to encompass these aspects.

A genetic dissection of invasive M3 *S. pyogenes* isolates revealed that they bore closer resemblance to M3 *S. pyogenes* isolates obtained from non-invasive pharyngitis, than to other invasive M3 *S. pyogenes* isolates [27]. Concordantly, SDSE traits conferring a tropism for bone and joint are probably unintended side effects from a bacterial point of view, and rather reflect adaptations to superficial tissues normally colonized and infected by SDSE. Moreover, both fibronectin and collagen are present in many different organs in the human body, including skin-tissue, and overlapping adhesive determinants for OAIs and skin and soft-tissue infections is conceivable. Such a notion is substantiated by several reports of patients presenting with two concomitant clinical entities, i.e. the co-existence of skin and soft-tissue infections and OAI [28, 29]. Taken together, this could partly account for the inability to segregate OAI isolates and non-OAI isolates based on their genomic content or phenotypic binding properties.

Our study is limited by the retrospective acquisition of clinical data. However, a meticulous review of medical records likely minimized diagnostic misclassification.

Secondly, although our genetic analyses indicate a correlation between the FCT-region and bacterial phenotypic binding abilities, we lack the experimental evidence to underpin our hypothesis. The possibility that the genetic content of the FCT-region represents a confounding variable cannot be ruled out, and our findings should be validated in subsequent studies. Moreover, knowledge on virulence determinants in SDSE is limited, and the predicted binding abilities of several of the putative adhesins presented herein, are extrapolated from properties of conserved domains in other bacterial species.

Thirdly, a subset of the OAI isolates included in our study were associated with recent joint surgery, suggesting per-operative introduction of SDSE into the joint cavity rather than haematogenous dissemination. Furthermore, several of the patients had orthopaedic implants composed of cobalt-chromium or titanium alloys, and the use of stainless steel as a representative of such implants has not been experimentally justified. This heterogeneity within the groups might have influenced the results, and obscured potential correlations to clinical entity.

Lastly, we acknowledge that the pathogenetic mechanism of OAI are multifactorial, and although adhesion to host surfaces is essential for the initiation of these infections, the interplay between the host immune response and the immune-evasive strategies of the pathogen are likely of equal importance. Exploration of these factors was beyond the scope of this study, but would undoubtedly have provided a more comprehensive knowledge on the disease processes involved. Nevertheless, we believe that our results constitute an important contribution to the overall understanding of the pathogenetic pathways of OAIs.

## Conclusions

We found the phenotypic binding abilities of SDSE to concord with genetic variations in their FCT-regions, but not with clinical manifestation. Our results also indicate that the increasing incidence of OAIs caused by SDSE in our region was not related to clonal expansion of particular strains. Hence, despite a thorough genetic and phenotypic dissection, the basis for the arthritogenicity of SDSE remains elusive.

## Methods

### Study population

The epidemiology of β-haemolytic streptococcal OAIs in western Norway from 1999 to 2013 has previously been described [4]. All available SDSE-isolates from this cohort were included in the study. The control group consisted of temporally and geographically matched SDSE blood culture isolates, obtained from patients without OAI, despite having one or more pre-existing local risk factors for developing OAI. Furthermore, the two groups were matched for presence and magnitude of systemic comorbidity by using the modified Charlson comorbidity score [30, 31]. Invasive disease, osteoarticular infections and other clinical entities were defined as previously described [4, 19]. Local risk factors comprised the presence of orthopaedic implants or osteosynthetic material, pre-existing joint disease or sequelae, or an invasive joint procedure performed within the past four weeks prior to admission.

### Bacterial isolates, DNA extraction and sequencing

All SDSE isolates included in the study displayed large colony size (> 0.5 mm in diameter after 24 h) and β-haemolysis on 5% sheep blood agar. Serogroup specificity and species identity was confirmed as previously described [32]. DNA was extracted using MagNA Pure 96 (Roche Life science, Basel, Switzerland). Genomic libraries were made using Nextera XT Kit (Illumina, Essex, UK), and 150 basepairs paired-end sequencing was performed on HiSeq 4000 (Illumina).

### Assembly, annotation and molecular characterization

The quality of the reads was checked with FastQC (bioinformatics.bbsrc.ac.uk), and subsequently filtered using Trimmomatic [33]. The Center for Genomic Epidemiology (CGE) website was used for online assembly by Spades [34]. The assemblies were assessed using Quast [35], and RAST was used for annotation [36]. *emm*-type and MLST-profiles were identified as previously outlined [26], and novel MLST alleles and profiles were submitted to pubMLST (www.pubMLST.org). SNP based phylogenetic analysis was performed [37], using *Streptococcus dysgalactiae* subspecies *equisimilis* ATCC 12394 (GenBank accession number CP002215) as a reference-genome. The presence and mutation of selected virulence genes and regions was examined using Geneious version 10.0 [38]. The genes and genetic regions investigated is presented in Table 2. Phylogenetic trees were constructed in Geneious by global alignment with free end gaps. The Tamura-Nei and the Jukes-Cantor models were used for calculating genetic distances in Figs 1 and 4, respectively. Trees were visualised and rendered using FastTree [39]. Identification of conserved domains in virulence proteins were performed using Conserved Domain BLAST (https://www.ncbi.nlm.nih.gov/Structure/cdd/wrpsb.cgi). To identify genes potentially associated with clinical category, a genome-wide association study was performed. First, the whole genome sequences were annotated with Prokka [40]. Subsequently the genetic repertoire of each isolate was analysed using Roary [41], and finally potential correlations between disease cohort and genetic content examined for statistical significance with Scoary [42]. *P*-values were adjusted using the Bonferroni correction method.

### Fibronectin and collagen binding assay

The binding assay was performed as previously described [32, 43], with some modifications. Human fibronectin (Fibrp-ro Roche, Sigma-Aldrich) was diluted in Dulbecco-Phosphate Buffered Saline (D-PBS) to 20 μg/ml, and human collagen type I and II (Catalogue no 1005 and 2015, Chondrex) was prepared according to the manufacturer’s instructions. Black 96-well microplates (Nunc Maxisorp, Thermo Scientific) were coated with 100 μl of fibronectin or collagen solutions in each well, and incubated overnight at 4 °C. The plates were subsequently washed three times with D-PBS, and blocked with StartingBlock™ (Catalogue no 37538, Thermo Scientific) as specified by the manufacturer. Uncoated microplates blocked with StartingBlock™ served as controls.

Bacterial isolates were grown overnight on 5% sheep blood agar in 5% CO2, and individual colonies were then suspended in 15 ml of Todd Hewitt broth (T1438, Sigma Aldrich) supplemented with 0.5% Yeast extract (Y0375, Sigma Aldrich) to an OD^600^ of 0.05. Suspensions were incubated in ambient air in a rotary shaker at 37 °C to the mid-log-phase (OD^600^ approximately 0.5), identified in preliminary experiments as the growth phase yielding the highest binding to fibronectin and collagen. Bacterial cells were harvested by centrifugation, washed three times, resuspended in D-PBS to 0.5 McFarland (~ 1 × 10^8^ Colony Forming Units [CFU]/ml), and 100 μl was added to each well of the coated microplates. A standard curve was constructed for each strain by inoculating 100 μl of the serial dilutions (~ 10^8^ CFU/ml, ~ 10^7^ CFU/ml and ~ 10^6^ CFU/ml) into separate wells. The plates were then centrifuged at 800 x *g* for 10 min and incubated at 37 °C for 30 min. The wells not containing the standards were washed three times with D-PBS, and subsequently refilled with 100 μl of sterile D-PBS. The DNA-binding fluorescent dye SYBR-Green I (S7563, Invitrogen) was used for quantification of bacterial adherence as previously described [32]. All experiments were performed in triplicates, and repeated thrice. Signal intensity obtained from the control-plates was subtracted, and the resulting data compared to the standard curves. The data are presented as the mean of all the measurements. To facilitate correlation of phenotypic binding abilities to genetic content, the isolates were dichotomized into strong and weak binders based on arbitrary cut-off values defined post-hoc, dividing the isolates into two approximately equally sized groups.

### Binding to stainless steel

Several different metal alloys are presently used in orthopaedic implants, whereof cobalt-chromium, stainless steel and titanium are the predominant. Among these, we chose stainless steel as a representative of osteosynthetic material, primarily due to pricing and availability. Special microplate lids were constructed, containing 96 stainless-steel grade SS316 nails mounted to fit the individual wells of a 96 well microplate. The nails were positioned to target the centre and bottom of each well at a 90° angle. The lid-constructs were thoroughly sanitized and sterilized prior to experimental use; submitted to ultrasonication for 15 min in a strongly alkaline commercial detergent, rinsed and boiled for 15 min in distilled water and finally sterilized by autoclavation. SDSE isolates were harvested from the mid-log phase as specified above, washed three times, and resuspended in D-PBS to 1.5 McFarland. 200 μl were added to individual wells of flat-bottomed 96 well microplates (Greiner, Austria), and D-PBS alone was inoculated into control wells. The microplates were mounted on a vortex shaker to keep the bacteria suspended, and incubated with the stainless-steel lid-constructs in ambient air at 37 °C for 1 h. The lids were subsequently removed, washed three times by submerging the nail-tips in D-PBS, and heat-fixated at 60 °C for 1 h. Quantification of adherent bacterial biomass was performed using the crystal violet technique as described elsewhere [44]. Optical densities at 600 nm were measured on a microplate reader (FluoStar Omega, BMG Labtech). The experiments were performed in triplicates, and repeated thrice. The mean values for each strain was divided by the mean values obtained from control wells in each experimental setup, and the strains were categorized as strong, medium, weak or non-binders as previously outlined [45].

### Statistical analysis

SPSS PASW Statistics, version 21.0 (IBM SPSS Statistics for Windows, Version 21.0. Armonk, NY: IBM Corp) was used for statistical processing. Non-parametric data were analysed with Mann-Whitney U-test, and categorical data were analysed using Fisher’s exact test. A two-sided *p*-value ≤0.05 was considered statistically significant.

## Additional file

Additional file 1: **Table S1** Overview of genotypic and phenotypic characteristics of the SDSE – isolates. Presents an overview of the *emm*-type, MLST-profile, FCT_1_-variant and binding properties associated with each of the 53 SDSE-isolates. (PDF 490 kb)

## Abbreviations

BLAST: Basic Local Alignment Search Tool
CFU: Colony Forming Units
CGE: Center for Genomic Epidemiology
D-PBS: Dulbecco-phosphate buffered saline
FCT-region: Fibronectin binding, Collagen binding and T-antigen region
MALDI ToF MS: Matrix-assisted laser desorption ionization time of flight mass spectrometry
MLST: multilocus sequence typing
OAI: Osteoarticular infection
SDSE: *Streptococcus dysgalactiae* subspecies *equisimilis*
SNPs: Single nucleotide polymorphisms

## References

[1] Ryan MJ, Kavanagh R, Wall PG, Hazleman BL (1997). Bacterial joint infections in England and Wales: analysis of bacterial isolates over a four year period. Br J Rheumatol.

[2] Murillo O, Grau I, Lora-Tamayo J, Gomez-Junyent J, Ribera A, Tubau F, Ariza J, Pallares R (2015). The changing epidemiology of bacteraemic osteoarticular infections in the early 21st century. Clin Microbiol Infect.

[3] Kennedy N, Chambers ST, Nolan I, Gallagher K, Werno A, Browne M, Stamp LK (2015). Native joint septic arthritis: epidemiology, clinical features, and microbiological causes in a New Zealand population. J Rheumatol.

[4] Oppegaard O, Skrede S, Mylvaganam H, Kittang BR (2016). Temporal trends of beta-haemolytic streptococcal osteoarticular infections in western Norway. BMC Infect Dis.

[5] Schattner A, Vosti KL (1998). Bacterial arthritis due to beta-hemolytic streptococci of serogroups a, B, C, F, and G. Analysis of 23 cases and a review of the literature. Medicine (Baltimore).

[6] Kurtz S, Ong K, Lau E, Mowat F, Halpern M (2007). Projections of primary and revision hip and knee arthroplasty in the United States from 2005 to 2030. J Bone Joint Surg Am.

[7] Green A, Christian Hirsch N, Pramming SK (2003). The changing world demography of type 2 diabetes. Diabetes Metab Res Rev.

[8] Seng P, Vernier M, Gay A, Pinelli PO, Legre R, Stein A (2016). Clinical features and outcome of bone and joint infections with streptococcal involvement: 5-year experience of interregional reference centres in the south of France. New Microbes New Infect.

[9] Shirtliff ME, Mader JT (2002). Acute septic arthritis. Clin Microbiol Rev.

[10] Xu Y, Rivas JM, Brown EL, Liang X, Hook M (2004). Virulence potential of the staphylococcal adhesin CNA in experimental arthritis is determined by its affinity for collagen. J Infect Dis.

[11] Johansson A, Flock JI, Svensson O (2001). Collagen and fibronectin binding in experimental staphylococcal osteomyelitis. Clin Orthop Relat Res.

[12] Ratcliffe E (2014). Staphylococcus aureus binding proteins for prevention of Orthopaedic implant-related infections. Microb Biochem Technol.

[13] Davies MR, McMillan DJ, Beiko RG, Barroso V, Geffers R, Sriprakash KS, Chhatwal GS (2007). Virulence profiling of streptococcus dysgalactiae subspecies equisimilis isolated from infected humans reveals 2 distinct genetic lineages that do not segregate with their phenotypes or propensity to cause diseases. Clin Infect Dis.

[14] Bisno AL, Craven DE, McCabe WR (1987). M proteins of group G streptococci isolated from bacteremic human infections. Infect Immun.

[15] Kreikemeyer B, Nakata M, Oehmcke S, Gschwendtner C, Normann J, Podbielski A (2005). Streptococcus pyogenes collagen type I-binding Cpa surface protein. Expression profile, binding characteristics, biological functions, and potential clinical impact. J Biol Chem.

[16] Falugi F, Zingaretti C, Pinto V, Mariani M, Amodeo L, Manetti AG, Capo S, Musser JM, Orefici G, Margarit I (2008). Sequence variation in group a streptococcus pili and association of pilus backbone types with lancefield T serotypes. J Infect Dis.

[17] Bessen DE, Kumar N, Hall GS, Riley DR, Luo F, Lizano S, Ford CN, McShan WM, Nguyen SV, Dunning Hotopp JC (2011). Whole-genome association study on tissue tropism phenotypes in group a streptococcus. J Bacteriol.

[18] Bowden MG, Heuck AP, Ponnuraj K, Kolosova E, Choe D, Gurusiddappa S, Narayana SV, Johnson AE, Hook M (2008). Evidence for the "dock, lock, and latch" ligand binding mechanism of the staphylococcal microbial surface component recognizing adhesive matrix molecules (MSCRAMM) SdrG. J Biol Chem.

[19] Oppegaard O, Mylvaganam H, Kittang BR (2015). Beta-haemolytic group a, C and G streptococcal infections in western Norway: a 15-year retrospective survey. Clin Microbiol Infect.

[20] Springman AC, Lacher DW, Waymire EA, Wengert SL, Singh P, Zadoks RN, Davies HD, Manning SD (2014). Pilus distribution among lineages of group b streptococcus: an evolutionary and clinical perspective. BMC Microbiol.

[21] Myhre EB, Kuusela P (1983). Binding of human fibronectin to group a, C, and G streptococci. Infect Immun.

[22] Speziale P, Raucci G, Meloni S, Meloni ML, Wadstrom T (1987). Binding of collagen to group a, B, C, D and G streptococci. FEMS Microbiol Lett.

[23] Reissmann S, Gillen CM, Fulde M, Bergmann R, Nerlich A, Rajkumari R, Brahmadathan KN, Chhatwal GS, Nitsche-Schmitz DP (2012). Region specific and worldwide distribution of collagen-binding M proteins with PARF motifs among human pathogenic streptococcal isolates. PLoS One.

[24] Bouchiat C, Moreau K, Devillard S, Rasigade JP, Mosnier A, Geissmann T, Bes M, Tristan A, Lina G, Laurent F (2015). Staphylococcus aureus infective endocarditis versus bacteremia strains: subtle genetic differences at stake. Infect Genet Evol.

[25] Tyrrell GJ, Lovgren M, St Jean T, Hoang L, Patrick DM, Horsman G, Van Caeseele P, Sieswerda LE, McGeer A, Laurence RA (2010). Epidemic of group a streptococcus M/emm59 causing invasive disease in Canada. Clin Infect Dis.

[26] Oppegaard O, Mylvaganam H, Skrede S, Lindemann PC, Kittang BR (2017). Emergence of a streptococcus dysgalactiae subspecies equisimilis stG62647-lineage associated with severe clinical manifestations. Sci Rep.

[27] Shea PR, Beres SB, Flores AR, Ewbank AL, Gonzalez-Lugo JH, Martagon-Rosado AJ, Martinez-Gutierrez JC, Rehman HA, Serrano-Gonzalez M, Fittipaldi N (2011). Distinct signatures of diversifying selection revealed by genome analysis of respiratory tract and invasive bacterial populations. Proc Natl Acad Sci U S A.

[28] Everts RJ, Chambers ST, Murdoch DR, Rothwell AG, McKie J (2004). Successful antimicrobial therapy and implant retention for streptococcal infection of prosthetic joints. ANZ J Surg.

[29] Osiri M, Akkasilpa S, Reinprayoon S, Deesomchok U (1996). Streptococcal arthritis in Thai adults: case series and review. J Med Assoc Thail.

[30] Charlson ME, Pompei P, Ales KL, MacKenzie CR (1987). A new method of classifying prognostic comorbidity in longitudinal studies: development and validation. J Chronic Dis.

[31] Hwang J, Chow A, Lye DC, Wong CS (2016). Administrative data is as good as medical chart review for comorbidity ascertainment in patients with infections in Singapore. Epidemiol Infect.

[32] Oppegaard O, Mylvaganam H, Skrede S, Jordal S, Glambek M, Kittang BR (2017). Clinical and molecular characteristics of infective beta-hemolytic streptococcal endocarditis. Diagn Microbiol Infect Dis.

[33] Bolger AM, Lohse M, Usadel B (2014). Trimmomatic: a flexible trimmer for Illumina sequence data. Bioinformatics.

[34] Nurk S, Bankevich A, Antipov D, Gurevich AA, Korobeynikov A, Lapidus A, Prjibelski AD, Pyshkin A, Sirotkin A, Sirotkin Y (2013). Assembling single-cell genomes and mini-metagenomes from chimeric MDA products. J Comput Biol.

[35] Gurevich A, Saveliev V, Vyahhi N, Tesler G (2013). QUAST: quality assessment tool for genome assemblies. Bioinformatics.

[36] Aziz RK, Bartels D, Best AA, DeJongh M, Disz T, Edwards RA, Formsma K, Gerdes S, Glass EM, Kubal M (2008). The RAST server: rapid annotations using subsystems technology. BMC Genomics.

[37] Kaas RS, Leekitcharoenphon P, Aarestrup FM, Lund O (2014). Solving the problem of comparing whole bacterial genomes across different sequencing platforms. PLoS One.

[38] Kearse M, Moir R, Wilson A, Stones-Havas S, Cheung M, Sturrock S, Buxton S, Cooper A, Markowitz S, Duran C (2012). Geneious basic: an integrated and extendable desktop software platform for the organization and analysis of sequence data. Bioinformatics.

[39] Price MN, Dehal PS, Arkin AP (2010). FastTree 2--approximately maximum-likelihood trees for large alignments. PLoS One.

[40] Seemann T (2014). Prokka: rapid prokaryotic genome annotation. Bioinformatics.

[41] Page AJ, Cummins CA, Hunt M, Wong VK, Reuter S, Holden MT, Fookes M, Falush D, Keane JA, Parkhill J (2015). Roary: rapid large-scale prokaryote pan genome analysis. Bioinformatics.

[42] Brynildsrud O, Bohlin J, Scheffer L, Eldholm V (2016). Rapid scoring of genes in microbial pan-genome-wide association studies with Scoary. Genome Biol.

[43] Jeng A, Sakota V, Li Z, Datta V, Beall B, Nizet V (2003). Molecular genetic analysis of a group a streptococcus operon encoding serum opacity factor and a novel fibronectin-binding protein, SfbX. J Bacteriol.

[44] Kwasny SM, Opperman TJ. Static biofilm cultures of gram-positive pathogens grown in a microtiter format used for anti-biofilm drug discovery. Curr Protoc Pharmacol. 2010;50:13A.8.1-13A.8.23. 10.1002/0471141755.ph13a08s50 PMC3272335 22294365

[45] Stepanovic S, Vukovic D, Hola V, Di Bonaventura G, Djukic S, Cirkovic I, Ruzicka F (2007). Quantification of biofilm in microtiter plates: overview of testing conditions and practical recommendations for assessment of biofilm production by staphylococci. APMIS.

