# Supplementary material for: Exploring the arthritogenicity of Streptococcus dysgalactiae subspecies equisimilis
Source: BMC Microbiol. 2018 Feb 27;18:17. doi: 10.1186/s12866-018-1160-5 (PMC5828338; doi:10.1186/s12866-018-1160-5)
Supplement: Supplementary file 1 — Table S1 Overview of genotypic and phenotypic characteristics of the SDSE – isolates. Presents an overview of the emm-type, MLST-profile, FCT1-variant and binding properties associated with each of the 53 SDSE-isolates. (PDF 490 kb) [file 12866_2018_1160_MOESM1_ESM.pdf]

Table S1 Overview of genotypic and phenotypic characteristics of the SDSE - isolates

Isolate identity	Clinical manifestation	*emm*-type	MLST-profile	FCT₁ variant	Fibronectin binding	Collagen I binding	Collagen II binding	Stainless steel binding
**T8**	OAI	*stG6.4*	335	FCT-6	33 %	9 %	5 %	2,3
**T55**	OAI	*stG6.3*	12	FCT-6	23 %	5 %	3 %	2,0
**T92**	OAI	*stG485.0*	29	FCT-6	51 %	0 %	0 %	3,1
**T108**	Pneumonia	*stG485.0*	29	FCT-6	35 %	0 %	0 %	2,0
**T113**	SSTI	*stG10.0*	15	FCT-6	5 %	73 %	74 %	2,8
**T114**	SSTI	*stG643.0*	12	FCT-6	9 %	5 %	4 %	3,3
**T148**	OAI	*stG643.0*	73	FCT-6	23 %	5 %	2 %	2,9
**T179**	Pneumonia	*stG485.0*	69	FCT-6	48 %	40 %	16 %	2,8
**T216**	SSTI	*stg480.0*	327	FCT-6	42 %	19 %	13 %	2,0
**T221**	OAI	*stg485.0*	55	FCT-6	77 %	2 %	1 %	2,3
**T256**	SSTI	*stg485.0*	29	FCT-6	37 %	1 %	0 %	2,4
**T261**	SSTI	*stg6.0*	63	FCT-6	19 %	0 %	1 %	4,9
**T274**	Pneumonia	*stG62647.0*	20	FCT-6	77 %	1 %	1 %	2,2
**T283**	SSTI	*stg6.0*	63	FCT-6	77 %	2 %	1 %	4,3
**T297**	OAI	*stg643.1*	328	FCT-6	28 %	27 %	27 %	2,7
**T299**	SSTI	*stc74a.0*	29	FCT-6	71 %	3 %	0 %	2,0
**T311**	SSTI	*stg485.0*	324	FCT-6	31 %	5 %	0 %	2,2
**T316**	SSTI	*stg6.1*	25	FCT-6	7 %	25 %	11 %	2,0
**T329**	SSTI	*stg652.0*	329	FCT-6	64 %	1 %	0 %	2,2
**T344**	SSTI	*stg643.0*	12	FCT-6	27 %	39 %	21 %	2,0
**T376**	NSTI	*stg485.0*	325	FCT-6	19 %	4 %	1 %	2,9
**T396**	OAI	*stG485.0*	29	FCT-6	52 %	0 %	0 %	2,7
**T399**	SSTI	*stg166b.0*	29	FCT-6	44 %	2 %	2 %	2,1
**T404**	OAI	*stG120.0*	323	**FCT-1**	22 %	82 %	68 %	2,4
**T412**	OAI	*stC36.0*	49	FCT-6	24 %	0 %	0 %	2,9
**T413**	OAI	*stg5420.0*	25	FCT-6	21 %	49 %	75 %	2,3
**T420**	OAI	*stg485.0*	128	FCT-6	42 %	30 %	13 %	2,3
**T429**	OAI	*stg643.0*	73	FCT-6	28 %	11 %	3 %	3,2
**T437**	OAI	*stG62647.0*	20	FCT-6	70 %	2 %	0 %	2,3
**T439**	Pneumonia	*stg480.0*	8	FCT-6	35 %	5 %	1 %	2,3
**T441**	OAI	*stg643.0*	12	FCT-6	5 %	10 %	6 %	3,5
**T446**	SSTI	*stg643.0*	331	FCT-6	11 %	5 %	2 %	3,1
**T450**	OAI	*stc74a.0*	29	FCT-6	48 %	22 %	13 %	2,3
**T466**	OAI	*stg480.0*	8	FCT-6	45 %	5 %	2 %	2,2
**T469**	OAI	*stG643.0*	12	FCT-6	26 %	8 %	4 %	3,2
**T483**	OAI	*stG485.3*	332	FCT-6	75 %	1 %	1 %	2,9
**T488**	SSTI	*stc74a.0*	29	FCT-6	35 %	1 %	1 %	3,2
**T505**	SSTI	*stc74a.0*	17	FCT-6	31 %	0 %	0 %	2,8
**T512**	OAI	*stg485.0*	128	FCT-6	37 %	9 %	5 %	2,1
**T518**	OAI	*stg485.0*	17	FCT-6	23 %	2 %	1 %	3,4
**T519**	OAI	*stG480.0*	8	FCT-6	52 %	39 %	29 %	2,1
**T528**	SSTI	*stg643.0*	12	FCT-6	22 %	9 %	3 %	2,7
**T532**	SSTI	*stg166b.0*	29	FCT-6	58 %	1 %	0 %	3,2
**T539**	OAI	*stg166b.0*	29	FCT-6	56 %	5 %	0 %	3,8
**T557**	OAI	*stG10.0*	333	FCT-6	9 %	51 %	51 %	3,4
**T560**	OAI	*stG62647.0*	20	FCT-6	59 %	1 %	0 %	2,3

From «Exploring the arthritogenicity of *Streptococcus dysgalactiae* subspecies *equisimilis*»
Oppegaard et al. 2018.

Table S1 - continued

Isolate identity	Clinical manifestation	*emm*-type	MLST-profile	FCT_1 variant	Fibronectin binding	Collagen I binding	Collagen II binding	Stainless steel binding
**T562**	OAI	*stG62647.0*	20	FCT-6	55 %	2 %	0 %	2,3
**T570**	SSTI	*stg652.0*	114	**FCT-5**	4 %	0 %	1 %	4,8
**T577**	OAI	*stG6.0*	63	FCT-6	21 %	2 %	1 %	4,1
**T578**	SSTI	*stG485.0*	326	FCT-6	24 %	0 %	0 %	4,4
**T579**	OAI	*stG6.0*	334	FCT-6	10 %	1 %	1 %	4,4
**T596**	OAI	*stc74a.0*	29	FCT-6	54 %	0 %	0 %	3,1
**T599**	OAI	*stG62647.0*	20	FCT-6	80 %	0 %	0 %	2,2

SDSE, *Streptococcus dysgalactiae* subspecies *equisimilis*; OAI, osteoarticular infection; SSTI, skin and soft-tissue infection; NSTI, necrotizing soft-tissue infection; MLST, Multilocus sequence typing; FCT, fibronectin, collagen and T-antigen-region.
FCT_1 variant refers to the nine structurally different FCT-regions characterized in *Streptococcus pyogenes* (1). The data for Fibronectin and Collagen type I binding represents the amount of adherent bacterial cells as a percentage of the original inoculum. Stainless steel binding is presented as the mean binding value divided by the mean value obtained from the control wells. Novel MLST-profiles are marked in red.

References

1. Falugi F, Zingaretti C, Pinto V, Mariani M, Amodeo L, Manetti AG, Capo S, Musser JM, Orefici G, Margarit I, Telford JL, Grandi G, Mora M. 2008. Sequence variation in group A Streptococcus pili and association of pilus backbone types with lancefield T serotypes. J Infect Dis 198:1834-41. doi:10.1086/593176.
